# Myocardial Infarction with Non-Obstructive Coronary Arteries: A Puzzle in Search of a Solution

**DOI:** 10.31083/j.rcm2311379

**Published:** 2022-11-09

**Authors:** Riccardo Scagliola, Jacopo Senes, Manrico Balbi

**Affiliations:** ^1^Cardiovascular Disease Unit, Department of Internal Medicine, IRCCS Ospedale Policlinico San Martino, University of Genoa, 16132 Genoa, Italy

**Keywords:** MINOCA, epicardial etiologies, microvascular etiologies, diagnostic tools, therapeutic strategies

## Abstract

**Background:**

The term myocardial infarction with non-obstructive coronary arteries (MINOCA), defines a puzzling event occurring in the absence of obstructive coronary artery disease on coronary angiography and without an overt potential cause. However, a practical diagnostic work-up is often difficult, due to the heterogeneous etiologies and pathophysiology of MINOCA. This review aims to provide a comprehensive overview focusing on epidemiology, etiopathogenesis, diagnostic tools and therapeutic strategies for subjects with MINOCA, in order to provide a prompt and accurate diagnostic work-up and an adequate therapeutic approach in this subset population.

**Methods:**

This educational review was carried out by following the standard methods of the Cochrane Collaboration and the PRISMA statement. The terms “MINOCA” OR (“myocardial infarction” AND (“non-obstructive” OR “non-obstructive”)) were searched in PubMed and Embase databases (in Title and/or Abstract) from 1st January 2003 until 31st May 2022.

**Results:**

Etiologic findings, clinical presentation and the degree of hemodynamic impairment play a pivotal role in defining the patient’s natural history and prognostic outcome, and may significantly impact on the decision-making strategies and therapeutic approaches.

**Conclusions:**

Despite further advances in diagnostic and therapeutic strategies, MINOCA remains a challenging conundrum in clinical practice. Clinicians should be aware of the different potential etiologies and pathogenic mechanisms of MINOCA, in order to carry out a comprehensive diagnostic work-up and implement a tailored therapeutic approach.

## 1. Introduction

The term myocardial infarction with non-obstructive coronary arteries (MINOCA), has been progressively used in the literature to define a distinctive subset of myocardial infarctions (MI), occurring when coronary angiography detects a non-obstructive coronary artery disease. Therefore, the definition of such a puzzling clinical event requires the contextual presence of: (i) the MI criteria, according to the 4th definition of MI proposed by the European Society of Cardiology in 2018 [1]; (ii) the detection of non-obstructive coronary lesions, including the presence of mild coronary atherosclerosis (stenosis <30%) or subcritical coronary lesions (stenosis ≥30% and <50%) in any infarct-related coronary angiography; (iii) the absence of other non-ischemic causes (such as myocarditis, pulmonary embolism, cardiac contusion or Takotsubo cardiomyopathy) or of ischemic conditions with no coronary involvement (as in case of oxygen supply-demand mismatch) [2]. This review aims to provide a practical overview focusing on epidemiology, etiopathogenesis, diagnostic findings, prognostic outcomes and therapeutic approach concerning subjects with MINOCA, in order to provide a prompt and accurate diagnostic strategy and therapeutic approach.

## 2. Methods

This educational review was carried out by following the standard methods of the Cochrane Collaboration and the PRISMA statement. Using preferred Reporting Items for Systematic Reviews and Meta-Analysis guidelines [3], the terms “MINOCA” OR (“myocardial infarction” AND (“non-obstructive” OR “non-obstructive”) were searched in PubMed and Embase databases (in Titles and/or Abstracts) from 1st January 2003 until 31st March 2022. All available high-quality resources written in English containing information on epidemiology, etiopathogenesis, clinical findings, diagnosis and therapeutic strategies for MINOCA were included in our research.

## 3. Results

**Fig. 1.**
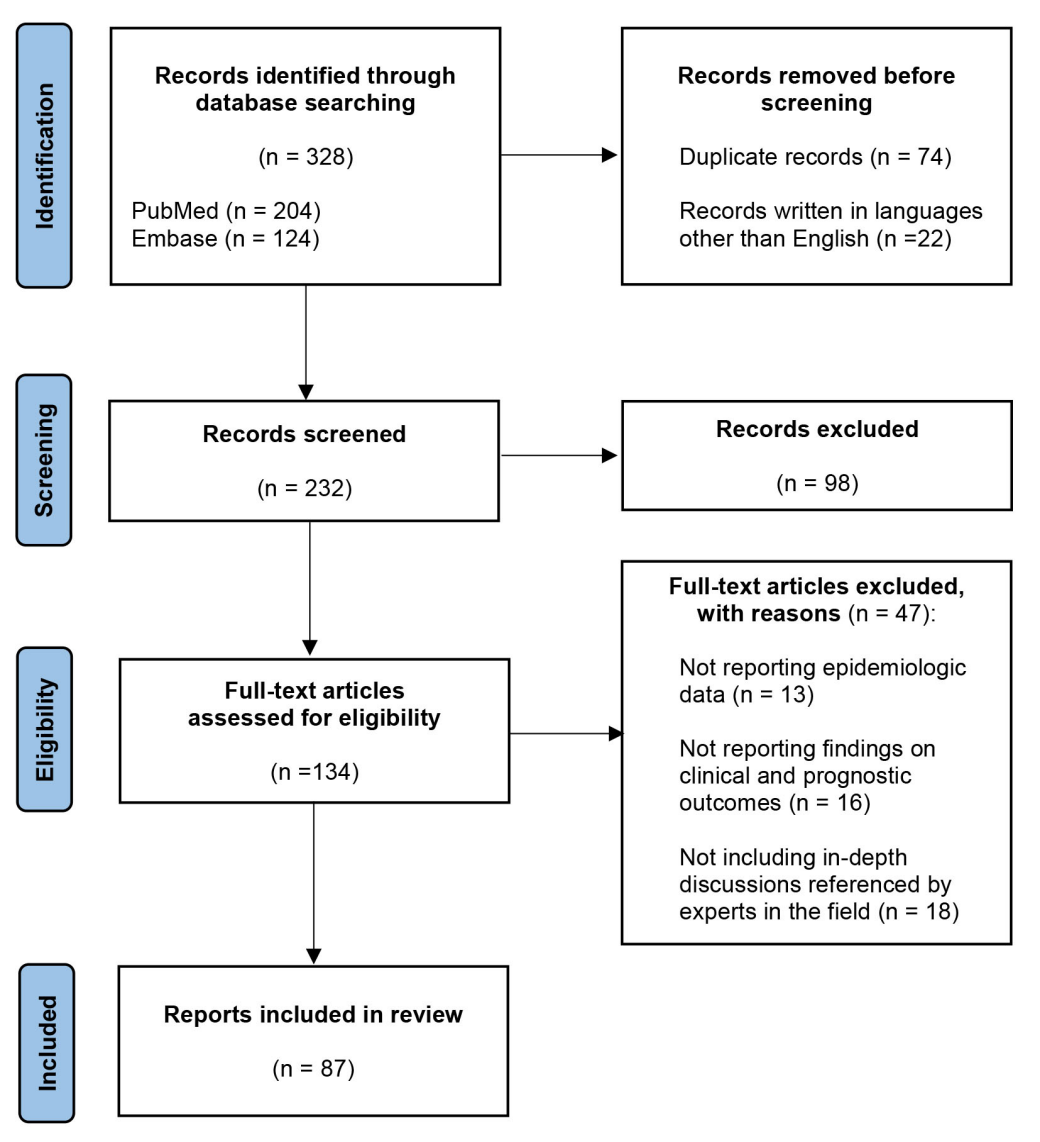
**Flow diagram**.

Out of the 328 records initially retrieved, 74 duplicates and 22 records in languages other than English were removed. Among the 232 remaining publications, 87 were included in our research material, on the basis of the following inclusion criteria: (a) peer-reviewed articles, (b) articles with abstract and full-text available, (c) articles reporting epidemiologic data, (d) articles reporting findings on clinical and prognostic outcomes, (e) articles including in depth discussions referenced by experts in the field (Fig. 1).

## 4. Epidemiology and Clinical Findings

Diagnosis of MINOCA has been reported in 5% to 15% of total subjects diagnosed with acute MI who undergo coronary angiography. The most recent studies conducted on general population cohorts of patients report a mean prevalence of 8.8%, although large MI registries attest to a value ranging from 5 to 25%, while its incidence ranges between 56,000 and 225,000 cases annually in the United States [4]. Cases of MINOCA are most common in the morning, and their incidence slightly increases in spring and autumn. Compared to patients with MI caused by “classical” obstructive coronary artery disease, subjects with MINOCA have been more frequently described as Afro-American females having a lower average age and fewer cardiovascular risk predictors, such as hyperlipidemia and diabetes [5]. Anxiety and depression seemed to be equally frequent among patients with MINOCA and obstructive MI, with a direct impact both on prognosis and quality of life. Despite the different etiologies of MINOCA, 12-lead electrocardiogram (ECG) can represent either ST-segment elevation or non-ST-segment elevation, with similar ratios in males and females. Furthermore, among patients with MINOCA, Bainey *et al*. [6] showed a significantly lower rate of cardiovascular re-hospitalization and one-year mortality, compared to subjects with MI related to obstructive coronary artery disease, although this difference declined in the long term.

## 5. Etiologies of MINOCA 

**Fig. 2.**
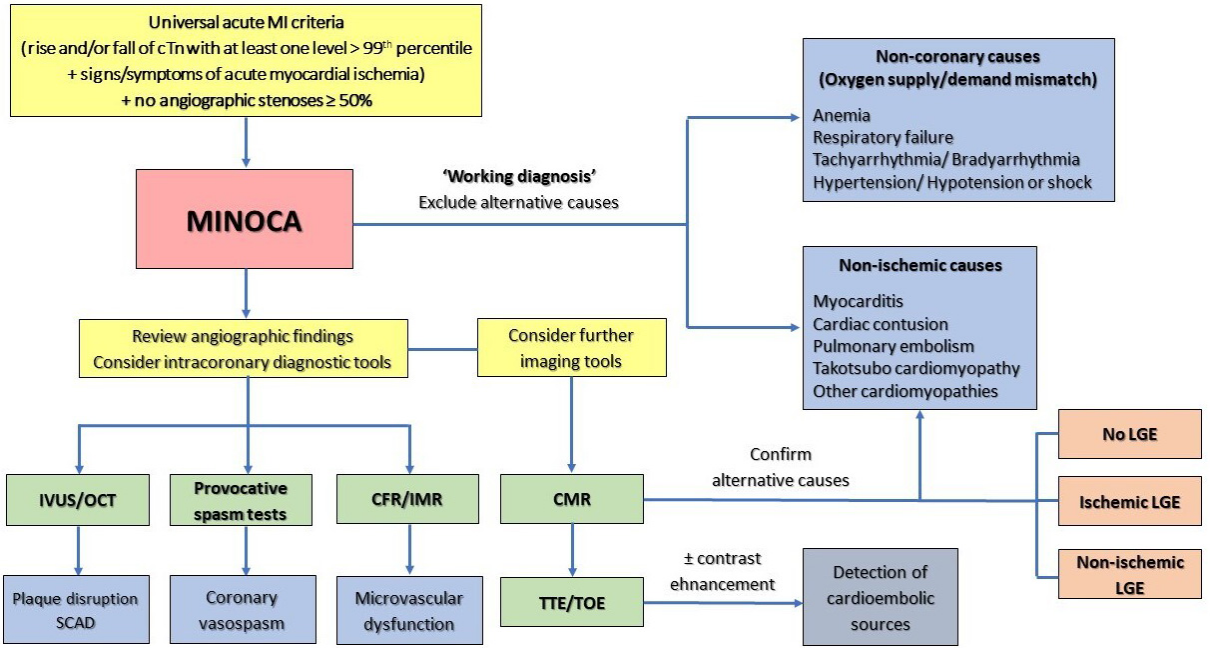
**Diagnostic flow-chart of patients presenting with suspected MINOCA**. CFR, coronary flow reserve; CMR, cardiac magnetic resonance; IMR, index of microvascular resistance; IVUS, intravascular ultrasound; LGE, late gadolinium enhancement; MI, myocardial infarction; MINOCA, myocardial infarction with non-obstructive coronary arteries; OCT, optical coherence tomography; TOE, transoesophageal echocardiography; TTE, transthoracic echocardiography.

**Fig. 3.**
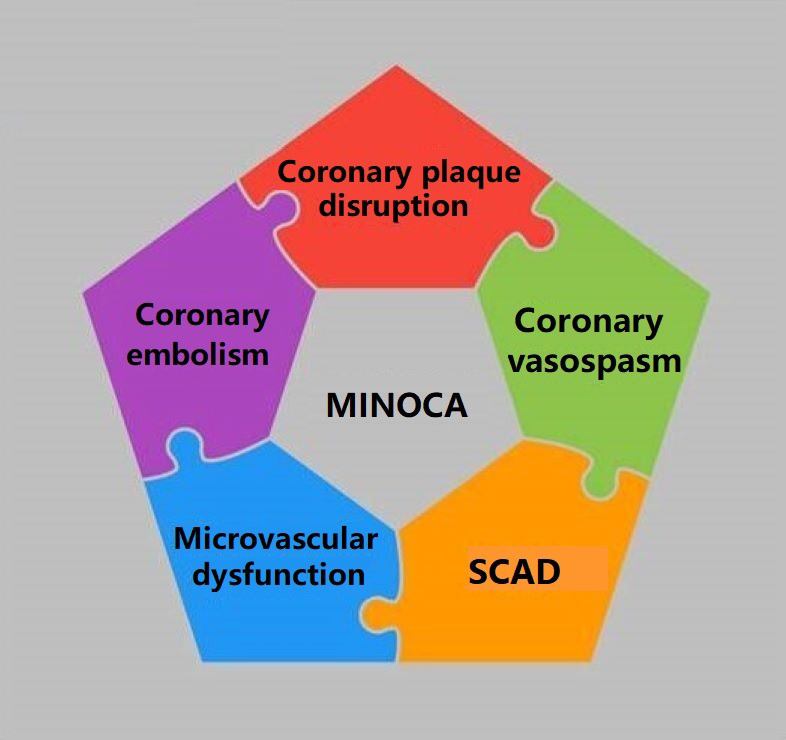
**Summary illustration including epicardial and microvascular etiologies of MINOCA**. MINOCA, myocardial infarction with non-obstructive coronary arteries; SCAD, spontaneous coronary artery dissection.

MINOCA is a working diagnosis, and it is necessary for physicians to investigate potential underlying causes, as failure to detect a specific underlying etiology may result in an inadequate therapeutic approach in these kinds of patients [2]. A diagnostic flow-chart of patients presenting with suspected MINOCA has been reported in Fig. 2. Several epicardial and microvascular causes of MINOCA have been identified, with potential overlaps between different etiologies (Fig. 3).

### 5.1 Epicardial Coronary Etiologies

#### 5.1.1 Coronary Plaque Disruption 

Coronary plaque disruption represents a relevant cause of MINOCA, involving 40% of total cases. In this critical context, acute thrombosis usually develops as a consequence of different pathophysiological processes, leading to a subcritical coronary stenosis rate (≤50%) [7]. The term ‘vulnerable plaque’ is usually used to define three main pathogenic lesions responsible for acute coronary thrombosis: plaque rupture, erosion, and eruptive calcified nodules [8]. Plaque rupture is the most frequent histopathological lesion leading to acute coronary syndrome. Ruptured atherosclerotic plaques are characterized by a discontinuity of their thin fibrous cap and a discontinuous intimal layer, together with a large necrotic core including lipids and inflammatory cells [9]. On the contrary, eroded plaques, which account for nearly 25% of ST-segment elevation MI, exhibit a thick, intact fibrous cap, with missing local endothelial cells, a smaller lipid core and a larger lumen area. Plaque erosions are characterized by apoptosis of the endothelium, with ensuing intimal denudation and exposure of pro-thrombotic agents. However, they typically maintain intact internal and external elastic laminas and have a well-represented tunica media with contractile smooth muscle cells; unlike ruptured plaques, which are characterized by a discontinuous internal lamina, together with a thin and less-developed tunica media [10]. Intracoronary imaging, particularly with high-resolution optical coherence tomography (OCT), plays a pivotal role in order to diagnose plaque rupture and to better characterize plaque morphology. In this regard, Dai and coworkers [11] described three different subsets of eroded plaques, based on plaque morphology: (i) fibrous plaque (defined as a lesion with a high backscattering and a homogeneous region); (ii) thick-cap fibroatheroma (characterized by a minimal fibrous cap thickness ≥65 μm); (iii) thin-cap fibroatheroma (defined by a minimal fibrous cap <65 μm). Finally, in 2–7% of overall acute coronary syndromes, eruptive calcified nodules are identified as a subset of MINOCA. They are characterized by a disruption of luminal surface by nodules of dense calcium with overlying thrombotic material, with no underlying necrotic core; they usually involve the intimal layer, the mid-right coronary artery and, most frequently, the left anterior descending coronary artery, at the sites of maximal torsion [12, 13]. Although the precise mechanism responsible for the formation of eruptive calcified nodules is still under investigation, a plausible hypothesis takes into account the increase in phosphate concentration in vascular smooth muscle cells and macrophage, which induces a shift towards a osteoblast-like-phenotype and promotes mineralization through the secretion of bone-associated proteins [14]. Although both ruptured and eroded plaques, as well as eruptive calcified nodules may give rise to potential acute thrombosis, the thrombus formed by plaque rupture generally consists of red thrombi, mainly formed by red blood cells and fibrin, while the surface of eroded plaques and eruptive calcified nodules is covered with white thrombi, mainly characterized by platelet and fibrinogen [9].

#### 5.1.2 Epicardial Coronary Vasospasm

Epicardial coronary spasm has been found in a wide range of patients with MINOCA, particularly in subjects under 50 years of age, with a prevalence rate of 16–74% of total cases, thus suggesting its pivotal role in the pathogenesis of acute myocardial ischemia in this subset population [5, 15]. An increasing prevalence of cases has been reported in women, particularly East Asians, especially from Korea and Japan [16, 17]. These demographic variations are partially related to genetic factors, such as the deficiency of the aldehyde dehydrogenase 2 genotipe variant, with consequent increase of toxic aldheyde levels in this subset of patients [18]. Several endogenous or exogenous vasoconstrictive triggers have been numbered in the literature, including smoking habits, cold exposure, psychological stress, hyperventilation, alcohol intake and stimulant agents (i.e., cocaine consumption) [19]. Chemotherapeutic agents (particularly belonging to the class of fluoropyrimidines) have been shown to induce endothelial injury and smooth muscle cell activation, with consequent myocardial ischemia secondary to epicardial coronary spasm [20, 21]. Furthermore, coronary vasospasm has been reported as a pivotal pathogenic mechanism involved in the Kounis syndrome, described as the occurrence of acute coronary syndrome triggered by an anaphylactic or anaphylactoid reaction [22, 23]. Finally, several studies have pointed out the occurrence of epicardial coronary spasm at segments with myocardial bridges, for which several pathogenic mechanisms have been hypothesized including myogenic myocardial mechanisms, abnormal coronary vasomotor mechanisms and impaired coronary adventitial vasa vasorum at the segments of the myocardial bridge [24]. Besides all the heterogeneous potential leading mechanisms, epicardial coronary spasm can be diagnosed in case of documented reduction of blood vessel diameter ≥75%, either occurring spontaneously or induced by pharmacological provocative testing with intracoronary acetylcholine (Ach), ergonovine or methylergonovine, together with clinical symptoms or instrumental findings of myocardial ischemia [5, 15].

#### 5.1.3 Spontaneous Coronary Artery Dissection (SCAD)

**Fig. 4.**
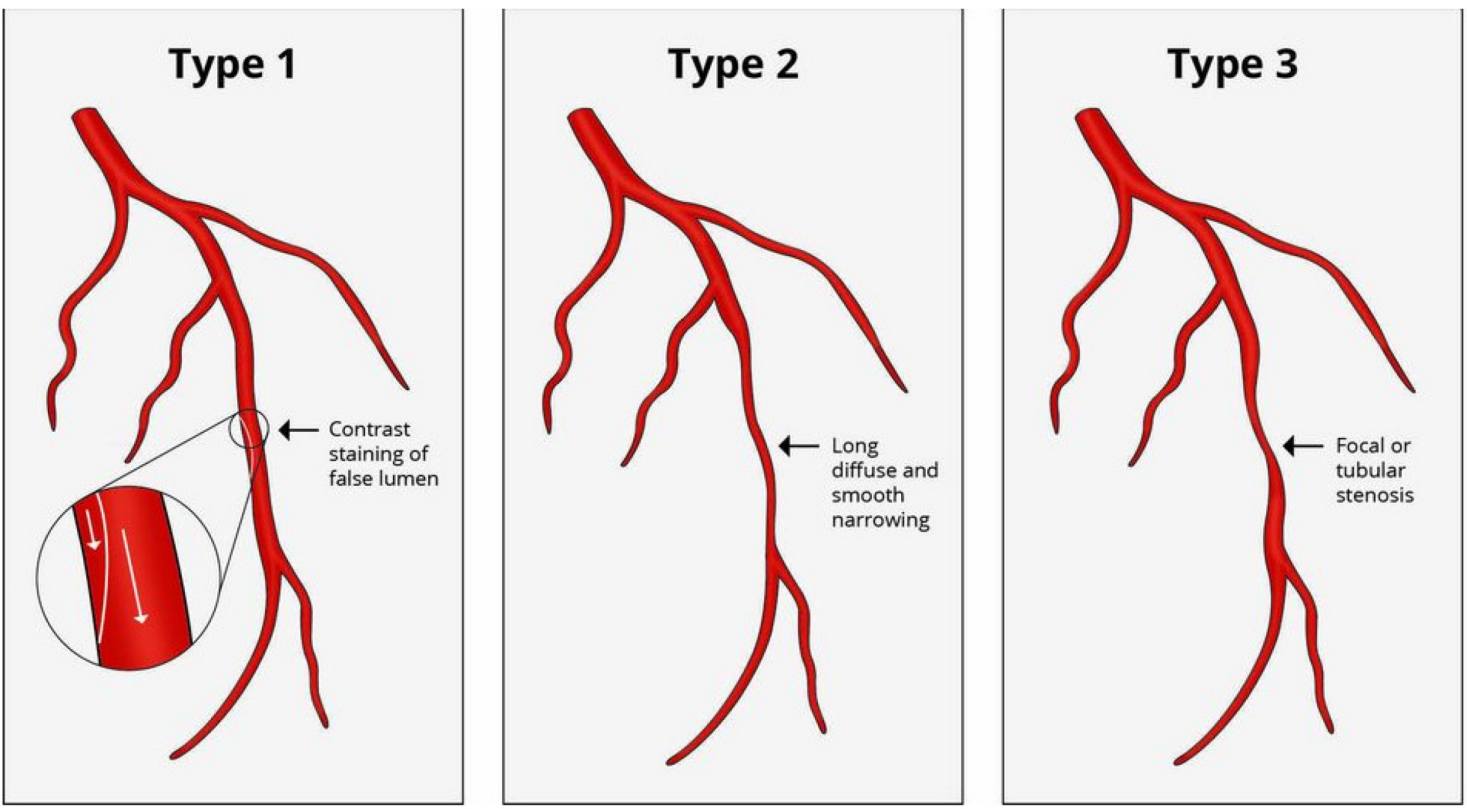
**Yip-Saw angiographic spontaneous coronary artery dissection**. Adapted from Teruzzi *et al*. [25].

SCAD represents another leading epicardial cause of MINOCA, with a mean prevalence rate of 4% among patients presenting with acute coronary syndrome, reaching a percentage of 35% in women under 50 years of age. Mechanisms of acute myocardial ischemia in SCAD refer to the development of an intramural hematoma within the tunica media, which predisposes to the separation from the underlying intimal layer and the compression of the true lumen [25, 26]. In accordance with the Yip-Saw angiographic classification, type 2 SCAD is the most common variant, and is characterized by a diffuse long smooth tubular lesion (typically >20 mm) because of a compressing intramural hematoma, with an abrupt change of the vessel caliper between normal and diseased segments. Specifically, type 2A SCAD has a normal vascular segment at its extremities, while type 2B SCAD prolongs up to the distal part of the vessel, and may appear as a ‘normal tapering’ vessel [27]. Type 1 SCAD consists of a longitudinal filling defect with contrast staining of the vessel wall and the appearance of double or multiple radiolucent lumens of different opacities [28]. Finally, type 3 SCAD is less frequent, due to multiple focal stenosis mimicking atherosclerosis. It is similar to type 2 SCAD, albeit shorter (usually <20 mm) and often requires intravascular imaging for the diagnosis [25] (Fig. 4, Ref. [25]). Several overlapping conditions may favor SCAD, including arterial hypertension, fibromuscular dysplasia, connective tissue diseases, inherited arteriopathies, systemic inflammatory conditions, as well as pregnancy. The latter takes a pivotal role in developing SCAD, particularly in the first weeks after delivery due to the hormonal and hemodynamic changes involved [29]. Changes in estrogen and progesterone levels drive structural changes in the tunica media, predisposing to coronary dissection (including impairment of connective tissue synthesis, increase in muchopolysaccharide content and fragmentation of elastic fibers) [25, 29]. On the other hand, anatomical factors, such as the presence of coronary tortuosity and the lack of intraluminal thrombus, predispose to SCAD recurrence and have a huge impact on prognostic outcomes [30]. Among diagnostic tools, OCT is the most accurate imaging technique in detecting SCAD and guiding coronary intervention, as it allows a better detection of both dissection length and changes in lumen diameter and because it is less affected by coronary calcifications than intravascular ultrasound (IVUS) [31].

### 5.2 Microvascular Coronary Etiologies

#### 5.2.1 Coronary Microvascular Dysfunction

**Fig. 5.**
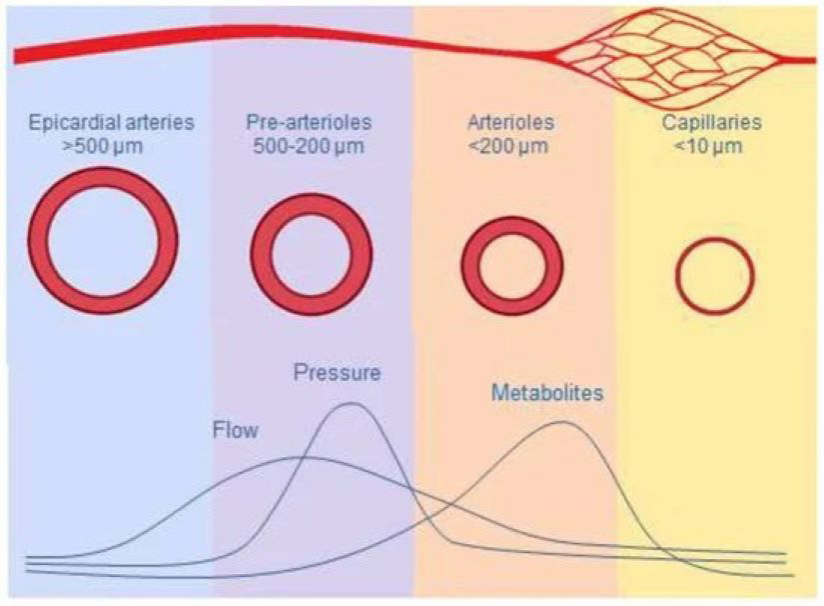
**Macro and micro coronary circulation and mechanisms inducing vasodilatation**. Adapted from Vancheri *et al*. [33].

Coronary microvascular dysfunction represents another leading cause of MINOCA, with a mean prevalence rate of 30% of patients with angina symptoms, particularly women with cardiovascular risk factors [32]. A standardized definition of coronary microvascular angina includes subjects with chest pain, together with the angiographic finding of non-obstructive epicardial coronary arteries, and an impaired coronary blood flow. The latter can be defined either as values of coronary flow reserve <2.0 or index of microcirculatory resistance ≥25 units after intracoronary vasodilator injection, or as the presence of coronary microvascular spasm diagnosed during intracoronary functional provocative test, or else as impaired coronary blood flow measured with a corrected Thrombolysis in Myocardial Infarction (TIMI) frame count [26, 33]. Endothelium has been shown to play a pivotal role in modulating vascular tone, due to its synthesis of endothelium-derived relaxing factors, including nitric oxide and vascular prostaglandins (which act mainly on epicardial coronary vasculature), and endothelium-dependent hyperpolarization factors, particularly hydrogen peroxide (which predominantly provokes vasodilatation of small resistance vasculature, such as coronary microvessels) [34]. As a result of this fine-tuned mechanism, increased myocardial metabolic activity promotes vasodilatation of the smallest arterioles (<40 μm), leading to the reduction of intraluminal pressure in medium-size arterioles (40–100 μm), which results in vasodilatation regulated by a myogenic response. This in turn increases flow upstream in the large arterioles (100–200 μm) through endothelium-dependent vasodilatation, in response to the wall share stress. Through these mechanisms, microcirculation regulates myocardial perfusion both at rest and at different levels of myocardial metabolic demands [35] (Fig. 5, Ref. [35]). Therefore, in this clinical setting endothelial-dependent microvascular dysfunction has been thought to be related to a reduced production of the aforementioned relaxing agents and their effects on coronary microcirculation. Furthermore, endothelium-dependent disfunction also involves microvascular inflammation and platelet activation, which in turn lead to vessel obstruction due to microvascular spasm, smooth cells proliferation and intimal thickening [32]. Endothelial cell dysfunction is also the pathophysiological key of coronary microvascular impairment detected in thrombotic microangiopathies. The latter include two typical phenotypes (thrombotic thrombocytopenic purpura and hemolytic uremic syndrome) and a spectrum of life-threatening clinical conditions, in which cardiovascular involvement is linked by a common pathophysiological basis, including endothelial damage of terminal arterioles and capillaries, with complete or partial microvascular occlusion caused by platelet and hyaline thrombi, and schistocyte formation due to the increased shear stress which impairs the membrane of red blood cells [36, 37]. Coronary microvascular spasm is defined as the concurrence of angina symptoms together with ECG abnormalities, without induction of coronary epicardial spasm during intracoronary pharmacologic provocative tests. Different pathogenic mechanisms of coronary microvascular spasm have been reported, including myosin light-chain phosporylation induced by Rho kinase and systemic inflammation leading to increased production of vasoconstrictive agents (i.e., serotonin or endothelin-1) and microvascular vasoconstriction [26, 38]. Additionally, also endothelium-independent vascular reactivity, unresponsive to intracoronary administration of adenosine, has been reported as the potential leading cause of myocardial ischemia due to coronary microvascular dysfunction, either related to atherosclerotic or non-atherosclerotic etiology [38]. Finally, intramural or extramural structural changes in the vascular wall (including luminal narrowing, vascular rarefaction, as well as extraluminal compression consequent to systemic disease) also contribute to coronary microvascular ischemia [39]. Specifically, a greater prevalence of cardiovascular risk factors (including diabetes mellitus, body overweight, dyslipidemia and older age) together with the presence of chronic inflammatory disorders, have been shown to promote inflammatory perivascular adipose tissue, which contributes to both epicardial and coronary microvascular flow disruption [40].

#### 5.2.2 Coronary Embolism

Albeit commonly reported in case series, coronary embolisms represent an infrequent and often unrecognized cause of acute coronary syndromes, affecting near 3% of patients with MINOCA, with a higher embolism recurrence and 10-year mortality rate, compared to non-embolic acute coronary syndromes [41]. Coronary arteries seem to be relatively protected from embolic sources, due to their acute angle takeoff from the aortic bulb, as compared to the aortic or distal systemic circulation. Three different types of embolic sources have been described: direct; paradoxical and iatrogenic, with potential overlap among them. Direct coronary emboli may originate from left sided thrombotic material. Left ventricular thrombotic material is usually reported as a consequence of coronary heart disease, while atrial fibrillation or mitral stenosis are common predisposing factors for thrombi located at the left atrium or left atrial appendage [42]. Infective endocarditic vegetations represent another underestimated cause of coronary embolism, which has been reported as embolic source in up to 60% of post-mortem assessments. Several risk factors for systemic embolization of infective vegetations have been proposed, including: (i) echocardiographic diameter greater than 10 mm); (ii) involvement of the mitral valve; (iii) staphylococcal or fungal infections [43]. Finally, albeit uncommon, cardiac tumors represent a predisposing source of coronary embolism, particularly villiform mixomas and papillary fibroelastomas [41, 44]. Furthermore, coronary embolism also includes the paradoxical migration of thrombotic material coming from the deep venous system and reaching the systemic circulation through a patent foramen ovale or atrial septal defect [45]. Finally, the presence of embolic material in the coronary circulation may be the consequence of interventional procedures (such as cardiac surgery or percutaneous interventions at the time of coronary or valvuloplasty procedure), particularly when systemic heparinization is not adequately maintained and catheters are not adequately flushed [46, 47]. Despite different causes, the common mechanism predisposing to coronary embolism is related to microvascular obstruction, leading to platelet cell activation and vasospasm, together with mechanical plugging of the microcirculation [32, 48].

### 5.3 Takotsubo Cardiomyopathy and the Unresolved Matter of Its Nosologic Framework

A proper nosologic framework for Takotsubo cardiomyopathy (TTC) remains an unresolved matter in clinical practice. The revised Mayo Clinic diagnostic criteria for TTC included: (i) the presence of transient left ventricular wall motion abnormalities (either hypokinesis, akinesis or dyskinesis) with or without apical involvement, (ii) usually extending beyond a single epicardial vascular distribution, (iii) in the absence of obstructive coronary artery disease on coronary angiography, (iv) associated with new ECG abnormalities or modest troponin increase, (v) in the absence of myocarditis or pheocromocytoma [2]. Subsequently, the following clarifications have been introduced by the International Takotsubo Diagnostic Criteria [49], in order to improve the identification of TTC: (a) subjects with wall motion abnormalities related to the distribution of a single epicardial coronary artery should not be considered an exclusion criteria for diagnosis of TTC; (b) pheocromocytoma, as well as neurologic disorders (i.e., ischemic stroke, transient ischemic attack or subarachnoid hemorrhage) are recognized as secondary causes of TTC; (c) the presence of contextual epicardial coronary lesions do not represent an exclusion criteria for diagnosing TTC. The latter supplemental findings together with the contextual detection of obstructive epicardial coronary disease make the classification of TTC as a distinct subset of MINOCA controversial. As reported by Lopez-Pais *et al*. [50] in a retrospective analysis on a large multi-center registry, TTC is often incidentally detected in subjects hospitalized for other extracardiac causes, and is characterized by a much more aggressive acute phase and by a better long-term prognostic outcome compared to the different subsects of MINOCA. Additionally, ECG findings like the absence of Q waves or reciprocal changes of ventricular repolarization, can help in distinguishing between TTC and MINOCA subjects [51]. Furthermore, the main pathophysiologic process responsible for developing reversible wall motion abnormalities in TTC differs from those related to the various subtypes of MINOCA, and seems to be related to the cathecolaminergic surge and the primary effect of norepinephrine spillover, mediated by both central and autonomic nervous system in response to psychophysical or environmental stressors [52]. This is reflected in a typical histopathological pattern called myocytolysis, which is characterized by early myofibrillar damage, hypercontracted sarcomeres and a mononuclear inflammatory response, compared to those noticed in MINOCA, which are instead characterized by myocytes without myofibrillar damage and polymorphonuclear infiltrates [53]. Finally, the presence of transient and reversible transmural myocardial oedema in the absence of late gadolinium enhancement (LGE) involving the dysfunctional wall segments at the cardiac magnetic resonance (CMR) is a pathognomonic hallmark for TTC, compared to MINOCA subsects [54]. Taken together, all these findings support the conceivable hypothesis that TTC could be defined as a unique pathologic entity rather than a distinct subsect of MINOCA. In this regard, further investigations are needed in order to define TTC with the most appropriate disease taxonomy.

## 6. Diagnostic Approach

Several diagnostic tools, including invasive and non-invasive diagnostic strategies are provided for the diagnosis of MINOCA, which remains a working diagnosis in order to better identify the underlying etiologic agents.

### 6.1 Non-Invasive Diagnostic Tools

#### 6.1.1 Echocardiography

Cardiac ultrasound is a first-level technique for assessing the causes of MINOCA. It should be performed in the acute onset, in order to identify ‘epicardial’ or ‘microvascular’ patterns by detecting regional wall motion abnormalities either involving a single epicardial coronary artery or extending beyond the myocardial wall region of a single epicardial coronary artery [2, 5]. Transthoracic and transoesophageal echocardiography also play a pivotal role in detecting sources of coronary embolism, such as ventricular thrombi, myxomas and papillary fibroelastomas and other cardiac tumors; valvular heart disease, endocarditis and unstable plaques in the ascending aorta. Furthermore, cardiac ultrasound may assess right-to-left interatrial shunts (demonstrated by i.v. microbubble infusion), thereby revealing the presence of patent foramen ovale, atrial septal defects or other intracardiac shunts [45].

#### 6.1.2 CMR

CMR imaging is a useful tool with patients with MINOCA, in order to confirm the diagnosis of MI and provide insights for detecting potential underlying causes. A prospective analysis by Pathik and co-workers [55] showed that CMR identified the underlying cause in 87% of patients with MINOCA. Particularly, CMR should be performed within 2 weeks after the onset of symptoms, in order to increase the diagnostic accuracy of the test in identifying the causes of MINOCA. Even very small necrotic areas can be detected, as the spatial resolution of CMR allows to detect even a mass as small as 0.16 g [56]. In subjects with MINOCA CMR may sometimes show large areas of myocardial oedema with or without small areas of necrosis, thus suggesting that coronary flow has been compromised transiently in a large vessel. This event may be attributed to a spontaneous coronary thrombolysis or to the occurrence of vasospasm. Finally, it allows LGE to be detected. CMR that reveals LGE allows to locate the area of myocardial damage and provides insight into the mechanisms of injury. For instance, an area of LGE in the subendocardium suggests an ischemic cause, though it cannot identify the cause of the ischemia (plaque disruption, vasospasm, thromboembolism, or dissection), while a sub-epicardial localization suggests cardiomyopathy. A non-ischemic appearance of LGE may suggest either myocarditis or an infiltrative disorder. CMR may be useful in the diagnostic work-up of MINOCA resulting from epicardial plaque disruption; in such cases, it enables the assessment of large areas of myocardial oedema and can detect transient flow compromission in a large vessel. It can also identify small well-defined areas of LGE, which suggests that atherothrombotic small vessel embolization from the site of disruption may be the main cause of myocardial necrosis in MINOCA patients [55]. Finally, in a subgroup of MINOCA patients, CMR is normal. This may be due to the fact that myocardial necrosis in these patients is too small to be detected. Alternatively, the normal CMR appearance may result from a wider spatial distribution of necrosis, i.e. necrotic myocytes may be scattered over a large area with no contiguous island of cell death of sufficient size to be detected by LGE imaging. Patients whose CMR is normal tend to display lower peak troponin values, though peak troponin values 100 times higher than the normal upper limit may occur even in patients who do not present LGE [56]. Furthermore, in patients with MINOCA and normal CMR, myocardial oedema imaging, which provides evidence of myocardial injury, is also absent. In the initial CMR studies, the finding of normality may be due to the fact that T2 imaging is undertaken late in the clinical course or that the CMR sequences utilized are not sufficiently sensitive [54, 56]. Developments in CMR techniques for imaging myocardial oedema and the routine application of CMR in MINOCA patients will provide further insights (Table 1).

**Table 1.** **Usefulness of cardiac magnetic resonance for diagnosis of MINOCA**.

	Myocardial oedema	Early gadolinium enhancement	Late gadolinium enhancement	Perfusion test	Distribution of gadolinium
Coronary plaque disruption	+/-	+	+	-	Subendocardial or transmural pattern
Coronary vasospasm	-	-	-	+	Transient and reversible perfusion defect with stress test
Spontaneous coronary artery dissection	+	+	+	-	Subendocardial or transmural pattern
Coronary embolism	+	+	+	-	Micro-macro embolization

+/-, capability of CMR techniques for the assessment of several forms of MINOCA.

#### 6.1.3 Screening for Inherited Thrombophilia

Studies on MINOCA patients have revealed that as many as 15% may have an abnormality that is detected on thrombophilia screening [57]. Several hypercoagulable disorders, including inherited or acquired causes, may predispose to a higher risk of coronary thromboembolic events. The main inherited causes of thrombophilia include factor V Leiden and increased levels of factor VIII, as well as protein C, protein S and factor XII deficiency, while acquired etiology may include antiphospholipid syndrome, heparin-induced thrombocytopenia, thrombotic thrombocytopenic purpura, autoimmune disorders and myeloproliferative tumors [58]. Although inherited thrombophilia may significantly impact on the increased risk for venous thromboembolism, no evidence of increased risk for arterial thromboembolism has been shown in long-term cohort trials of subjects with inherited thrombophilia. Furthermore, in patients with no known environmental and acquired thrombogenic factors and without long-term oral anticoagulation, the recurrence rate of thromboembolic events was near 10% on follow-up, thus suggesting a false reassurance for negative thrombophilia screening tests, because only a limited subset of thrombophilia mutations were taken into account [42].

#### 6.1.4 Coronary Computed Tomography (CT) Angiography

Coronary CT angiography has been progressively employed as a non-invasive diagnostic tool capable to provide a three-dimensional reconstruction set, allowing further geometrical characterization of cardiac chambers and epicardial coronary anatomy (albeit limited by increased heart rate, more than 70 beats per min). However, only a few data reported in the literature have investigated its systematic use in detecting underlying coronary atherosclerosis and critical coronary lesions in patients with MINOCA, also due to its low positive predictive value and diagnostic accuracy, particularly in distinguishing between coronary plaque rupture or erosion. For these reasons, the role of coronary CT angiography is still confined to excluding a low diagnostic suspicion of coronary atherosclerosis in subjects with a low pre-test probability, also because of its high specificity index [59]. Taken together, these findings suggest a still limited role of coronary CT angiography in diagnosis and therapeutic decision making for subjects with MINOCA.

### 6.2 Invasive Diagnostic Tools

#### 6.2.1 Coronary Angiography

The role of coronary angiography is the cornerstone for diagnosing MINOCA, as it allows to ascertain the absence of obstructive atherosclerotic lesions. However, unfortunately this procedure may sometimes result in a misleading diagnosis, owing to the fact that intravascular ultrasound studies have frequently demonstrated a significant atherosclerotic burden even in patients with “normal” coronary angiography [2]. Furthermore, the angiographic criteria for ‘non-obstructive coronary arteries’ detailed in the MINOCA definition utilize the conventional cut-off of >50% stenosis, which is consistent with the current angiographic guidelines. This conventional threshold is rather arbitrary and there is substantial inter- and intra-observer variability in the visual estimation of angiographic stenosis. Moreover, the dynamic pathophysiological nature of an acute coronary syndrome may result in significant angiographic changes arising from fluctuating coronary vasomotor tone and unstable coronary plaques (including a shifting thrombotic mass, plaque hemorrhage and washout of plaque contents) [5, 8]. Finally, coronary angiography cannot demonstrate, but only suggest, the presence of coronary plaque disruption in the presence of haziness or of a small filling defect; conversely, OCT or, to a lesser extent, IVUS should be used to identify plaque disruption [34]. Furthermore, coronary angiography may also play a role in the angiographic suspicion of coronary embolism, which may angiographically appear as a heavy thrombotic burden and filling defects in different coronary arteries, although bystander atherosclerotic lesions are commonly present, making angiographic diagnosis more challenging [42].

#### 6.2.2 Intracoronary Imaging Tools

Intracoronary imaging tools play a pivotal role in confirming non-obstructive coronary lesions in patients with acute coronary syndromes and identifying the various possible causes of MINOCA. Specifically, intracoronary imaging techniques have proved capable of identifying not only coronary plaque disruption (which encompasses plaque rupture, erosion and eruptive calcified nodules), but also its underling atherogenic mechanisms and consequent therapeutic approach. While thrombi are frequently detected at the site of plaque rupture, they cannot be found at the site of old ruptured atherosclerotic plaques, nor in the case of freshly ruptured plaques that have been promptly treated with anti-thrombotic therapies [2]. Thus, if executed at the time of cardiac catheterization, intracoronary imaging with either IVUS or OCT may be useful in identifying the most important causes of MINOCA. OCT should be preferred to IVUS, as it allows the identification of ruptured atherosclerotic plaque with thrombosis [2, 9]. Taking into account the current intracoronary imaging modalities, only OCT has been demonstrated to successfully identify plaque erosions, and distinguish between ‘defined’ and ‘probable’ OCT erosions (as the former consist of an unrupted fibrous cap and overlying white thrombus, while the latter are characterized by the absence of luminal thrombus or attenuation of the atherosclerotic plaque underlying the thrombus). Finally, although eruptive calcified nodules were first described by means of IVUS, OCT has proved superior in detecting them, as it shows fibrous cap breakage and/or thrombus over a calcified plaque protruding into the coronary lumen [3, 60]. Intracoronary imaging diagnostic tools may also help to detect coronary artery dissections. IVUS is a safe, accurate and reproducible imaging tool for detecting vessel wall structure; it can help differentiate between true and false coronary aneurysms, and allows intravascular assessment of coronary mural hematoma [31, 61]. However, OCT has been reported to be superior for the detection of coronary dissection, particularly in the presence of type 1 SCAD (in which a false lumen is detected), due to its higher spatial resolution and capability of generate high-resolution cross-sectional images of coronary wall structure [62]. Moreover, it allows a better definition of intimal flaps and can aid the assessment of guidewire location prior to percutaneous intervention. Finally, because of its greater diagnostic power in detecting intima-media layers and intramural hematoma, OCT plays a role in case of diagnostic uncertainty, especially for the diagnosis of type 3 SCAD, which either mimics atherosclerotic lesions or describes an abrupt vessel occlusion [25, 63] (Table 2). On the basis of the aforementioned properties of OCT, Taruya and coworkers investigated the potential impact of lesion characteristics on prognostic outcome in subjects with MINOCA. They found that nearly half of them were characterized by the presence of hidden ‘high-risk’ vascular wall lesions (i.e. ruptured or eroded plaques, calcified nodules, SCAD or endothelial dysfunction), and resulted in poorer outcomes than those affected by functional etiologies (i.e., coronary spasm) [64]. These findings underline the need to introduce intracoronary imaging tools in diagnostic flow charts for MINOCA, in order to rule out potential underlying organic causes and perform a proper risk stratification of MINOCA patients, particularly for those with mild coronary stenosis.

**Table 2.** **Comparison of intracoronary diagnostic tools for different etiologies of MINOCA**.

	FFR	IVUS	OCT	Main data
Coronary stenosis <50%	+	-	-	FFR negative for values <0.80
Coronary plaque rupture	-	+	++	Discontinuity of the fibrous cap and consequent distal embolization of its high lipidic necrotic core
Coronary plaque erosion	-	-	++	OCT is able to determine the so called “determined/probable” OCT plaque erosion
Eruptive calcified nodules	-	+	++	OCT better notices the protrusion of eruptive calcified nodules in vascular lumen
Thrombus	-	+	++	OCT is able to detect thrombotic components and distinguish between red or white thrombus
Spontaneous coronary artery dissection	-	+	++	OCT has a better capacity in determining intimal tears, false lumen
External vessel structure	-	++	+	IVUS has a lower spatial resolution, albeit a deep penetration in assessing external elastic lamina, compared to OCT

FFR, fractional flow reserve; IVUS, intravascular ultrasound; OCT, optimal coherence tomography. +/-, capability of intracoronary diagnostic tools for the assessment of the of coronary plaque disruption.

#### 6.2.3 Intracoronary Provocative Spasm Tests

In patients with MINOCA, if clinical data suggest coronary artery spasm, provocative testing by means of intra-coronary Ach or ergonovine should be performed in the diagnostic work-up. Provocative spasm testing has been seen to induce spasm in 27% of MINOCA patients, suggesting that it is a common and important pathogenic mechanism in MINOCA [3, 15]. Given that nitrates, and especially calcium channel blockers, are effective therapies for coronary artery spasm, and that the latter have been shown to prevent cardiac events in vasospastic angina, the diagnosis and treatment of coronary artery spasm need to be carefully considered [65]. Microvascular spasm is another potential cause of MINOCA, since elevated troponins have been detected via ultrasensitive assays following provocative spasm testing, despite the absence of inducible large-vessel spasm [66]. In this pathophysiologic context, further insight into the general safety and prognostic value of provocative spasm tests in MINOCA have been investigated. Data from the AChPOL Registry including patients undergoing intracoronary provocative test with Ach from December 2010 to March 2013 for a suspicion of variant angina or coronary microvascular spasm, showed a general safety and feasibility of intracoronary Ach use. Furthermore, over a median follow-up of 56 months, a significantly higher rate of recurrent chest pain requiring hospitalization has been reported in the microvascular spasm subgroup, compared to patients with negative intracoronary Ach test [67]. In a single-center analysis by Montone *et al*. [68] focusing on the role of abnormal coronary vasomotion as a trigger of acute coronary syndrome, the following findings were reported: (i) in MINOCA patients, provocative tests for spasm identify a large proportion of patients who would otherwise be discharged from hospital without a sure pathogenic diagnosis; (ii) provocative tests for spasm have prognostic significance; (iii) spasm can be safely elicited in the catheter laboratory even in the acute or subacute phases (i.e., within the first 48 h) of MINOCA. The safety of performing intracoronary provocative spasm tests in the acute setting of MINOCA has been confirmed in a systematic review conducted by Ciliberti *et al*. [69], who evaluated the safety of pharmacological provocative intracoronary tests with Ach or ergonovine in more than 9400 patients presenting with acute coronary syndrome or stable coronary artery disease. No deaths were reported and the overall occurrence of major (0.8%) and minor (4.7%) complications was low [70]. The most prevalent major complications included: ventricular tachycardia or ventricular fibrillation (0.69%), cardiogenic shock (0.03%), acute MI (0.01%), coronary dissection (0.01%), cardiac tamponade (0.01%) and prolonged spasm (0.01%). The most common minor event (2.17%) was the induction of marked bradycardia or transient second- or third-degree atrioventricular block following Ach injection into the right coronary artery, with spontaneous resolution within 3–5 s in the absence of associated symptoms [67, 68]. Taken together, these findings encourage interventional cardiologists to incorporate intracoronary provocative spasm tests in their routine clinical practice, as they may improve interventional strategies in subjects with MINOCA due to functional etiologies, and contribute to design a prompt diagnostic work-up and therapeutic approach in this subset population [71].

## 7. Prognostic Outcomes

Few studies have investigated the clinical outcome and in-hospital mortality of patients with MINOCA. The observational analysis conducted by Nordenskjöld *et al*. [72] on a large cohort of subjects recorded between July 2003 and June 2013, showed that independent predictors for new major cardiovascular events and death in MINOCA patients were somewhat similar to those reported for MI due to obstructive coronary artery disease. They include: older age, arterial hypertension, current smoking, diabetes mellitus, impaired renal function, and reduced left ventricular ejection fraction, as well as previous MI, previous stroke and peripheral vascular disease [72]. Additionally, Ciliberti and colleagues [73] showed that also the number of epicardial vessels affected by mild coronary artery disease (resulting in stenosis between 30% and 50%) and increased C-reactive protein concentrations, are markers of a worse prognostic outcome in this subset population. Data related to long-term prognostic outcomes in subjects with MINOCA are still limited. However, a metanalysis conducted by Pasupathy and colleagues [74] involving more than 55,360 MINOCA patients, revealed a significantly lower 12-month all-cause mortality compared to subjects with MI and obstructive coronary artery disease, and a statistically non-significant a trend toward a worse 12-month prognostic outcome compared to non-MI subjects. This metanalysis also reported a limited prognostic impact of atherosclerotic burden at 12-month prognosis, assessed by intracoronary imaging [74]. The association between the other pathogenic mechanisms of MINOCA and long-term prognostic outcomes in these patients have yet to be confirmed by broad multi-center prospective investigations. Furthermore, in subjects with non-obstructive coronary artery disease abnormal non-invasive stress tests may indicate the presence of myocardial ischemia in these patients. However, a diagnosis of myocardial ischemia based only on the positivity of non-invasive stress tests does not allow to stratify MINOCA patients on the basis of the risk of long-term cardiovascular events. Intracoronary imaging or provocative spasm tests, as well as invasive assessment of coronary microvascular dysfunction, have shown to enabling the identification of a subgroup of patients with a high-risk of long-term cardiovascular events and a worse prognosis [64, 75]. These findings reinforce the need of introducing intracoronary imaging and functional tests in daily clinical practice, in order to achieve an etiologic diagnosis of MINOCA, and to allow a prognostic stratification of these patients.

## 8. Therapeutic Strategies

Although evidence-based guidelines support coronary revascularization as the cornerstone for the treatment of patients with acute coronary syndromes and obstructive coronary lesions, data for patients with MINOCA are lacking. In this clinical context, a proper etiology-based therapeutic approach remains a major untackled issue for the management of MINOCA. In patients with coronary plaque disruption, double antiplatelet therapy is needed, despite percutaneous coronary intervention [26]. Furthermore, in order to reduce the atherogenic burden in this subset population, an aggressive management of all modifiable risk factors and the hypolipidic treatment with statins are of paramount importance [33, 40]. On the other hand, for subjects with MINOCA caused by either epicardial or microvascular coronary vasospasm, the first-line therapy of choice should consist of calcium channel blockers, which are able to reverse coronary spasm, whether epicardial or microvascular. Their administration should be started as soon as possible, especially in the event of life-threatening ventricular arrhythmic complications. Alternatively, nitrates may be used, owing to their ability to improve symptoms [32]. Studies conducted in Japan have shown that fasudil, a rho-kinase inhibitor which exerts strong coronary vasodilatation in the presence of epicardial coronary artery spasm, can also be used [76]. Beta-blockers, alone or in combination with vasodilators, may be useful. However, the administration of these drugs must be cautiously undertaken, owing to the fact that they trigger coronary vasoconstriction by indirectly stimulating coronary alpha-adrenoceptors [77]. Furthermore, to date the administration of beta-blockers together with angiotensin-converter enzyme inhibitors and statins is the cornerstone for the treatment of patients with MINOCA and coronary microvascular dysfunction with negative intracoronary provocative spasm tests [33]. The therapeutic approach of SCAD is currently a matter of extensive debate. Although double antiplatelet therapy (DAPT) remains the mainstay of the guideline-based approach for acute coronary syndromes, the post-procedural outcomes in SCAD are less predictable than acute coronary syndromes related to atherosclerotic lesions, due to a higher rate of iatrogenic dissections, abrupt vessel occlusion and hematoma propagation, the latter occurring in up to one third of total cases [25, 48]. Furthermore, data from the literature show a complete angiographic healing, often within 30 days after conservative pharmacological treatment [78]. For these reasons, recent evidences have pointed out a strategy ‘as conservative as possible’, thus reserving an interventional approach in case of patients with SCAD resulting in proximal coronary occlusion, or for patients with unstable hemodynamic, major ventricular arrhythmias or SCAD recurrence after medical therapy alone. As for patients treated conservatively, there is a lack of consensus about the use and duration of doubled antiplatelet therapy. Although the guideline-based therapy is one year, the pathophysiological mechanism of SCAD is different from that of coronary dissection related to atherosclerotic lesions, and in the former case a prolonged DAPT duration for subjects treated medically could cause potential bleeding within intramural hematoma, thus leading to the extension of coronary dissection and increased poor prognostic outcome [30]. For these reasons, Hayes and coworkers [25] have suggested a recommended DAPT duration of at least 2 to 4 weeks after the occurring SCAD episode, and the extension of low-dose aspirin administration for a period ranging from 3 to 12 months, thus encompassing the timeframe for SCAD healing. The final decision about extending the duration of antiplatelet therapy in this subset population, should consider several factors, including the patient’s bleeding risk and features predisposing to SCAD recurrence (including fibromuscular dysplasia, coronary tortuosity, undertreated arterial hypertension and history of dissections involving other systemic vascular territories) [29]. Finally, in the presence of coronary embolism, mechanical thrombectomy with aspiration has resulted in a significant reduction in cardiovascular death among selected patients with a high thrombotic burden. Aspiration of thrombotic material allows a better detection of underlying coronary arteries, and the subsequent application of IVUS or OCT may further assess even the presence of isolated subtle coronary plaque disruptions [9, 62]. Aspirated material deserves histological analysis, as it allows to distinguish between the presence of platelet and/or fibrin (which would be consistent with left heart or paradoxical right sided thrombus) and, less frequently, an embolic source from neoplasms or infected material, which would be directed to a specific therapeutic approach. In case of incomplete vascular reperfusion and partial thrombus removal, intracoronary thrombolytic agents such as the infusion of unfractionated heparin, bivalurudin or GpIIb/IIIa agents, may be considered in the management of distal embolization, albeit often with only partial benefit [79, 80]. The etiologic source of coronary embolism deserves an accurate diagnostic work-up: as highlighted by the current international guidelines, patients with diagnosed atrial fibrillation and systemic thromboembolism should be offered life-lasting oral anticoagulation, while the duration of the treatment with oral anticoagulants in patients with left-sided cardiac embolic source is currently a matter of debate. On the other hand, patients presenting with paradoxical coronary embolism of suspected venous origin should undergo a proper work-up including the assessment of patent foramen ovale and arteriovenous malformations [81, 82]. Patients with paradoxical coronary embolism and right-sided or major pulmonary embolism are associated with a 10-fold increased risk of mortality in retrospective series, compared with those without a paradoxical embolic origin. In this critical context, closure of patent foramen ovale and atrial septal defects by percutaneous devices have shown a significant long-term clinical benefit, compared to medical treatment alone [41].

## 9. Conclusions

Despite further advances in diagnostic and therapeutic strategies, MINOCA remains a challenging conundrum in clinical practice, in which several potential etiologies and pathogenic mechanisms may be identified, each of them requiring a tailored diagnostic work-up and therapeutic strategy. Clinicians should be aware of such a heterogeneous clinical entity, in order to carry out a comprehensive diagnostic strategy and a proper etiology-related therapeutic approach.

## Supplementary Material

Supplementary material associated with this article can be found, in the online version, at https://doi.org/10.31083/j.rcm2311379.
